# Efficacy and adverse effects of three delivery methods for aerosolized salbutamol in anesthetized European moose (*Alces alces alces*): a case series

**DOI:** 10.1186/s13028-026-00856-7

**Published:** 2026-02-08

**Authors:** Jacopo Morelli, Stefan Hoby, Elisabeth Heiderich, Marion Stettler, Dany Elzahaby, Alina L. Evans

**Affiliations:** 1 University of Inland Norway, Anne Evenstads Vei 80, Koppang, 2480 Norway; 2 Skeldale 24h Hospital (Medivet Thirsk), Lumley Close, Thirsk, YO7 3TD UK; 3 Bern Animal Park, Tierparkweg 1, Bern, 3005 Switzerland; 4 Anaesthesiology and Pain Therapy Section, Department of Clinical Veterinary Medicine, Vetsuisse Faculty, University of Bern, Hochschulstrasse 4, Bern, 3012 Switzerland

**Keywords:** *Alces alces alces*, Anesthesia, BMI, Butorphanol, European moose, Hypoxemia, Inhaler, Ketamine, Medetomidine, Salbutamol

## Abstract

**Background:**

Wild and captive European moose (*Alces alces alces*) are routinely anesthetized with drug combinations including alpha-2 adrenoceptor agonists, dissociative anesthetics, and opioids. Reportedly, severe respiratory depression, ventilation/perfusion (V/Q) mismatch, and hypoxemia are common complications in this species, requiring perianesthetic oxygen supplementation as key treatment, athough high flow rates can exacerbate respiratory acidosis. Salbutamol is a β_2_-adrenoceptor agonist effective in alleviating V/Q mismatch and hypoxemia in anesthetized horses when administered via endotracheal tube. Administration of salbutamol could elicit similar effects in anesthetized moose, improving animal welfare and perhaps reducing or replacing the supplemental oxygen requirements.

**Case presentation:**

Three healthy captive moose (two juveniles, male and female, and one adult male) underwent four anesthetic events for routine zoo health assessments. One juvenile was anesthetized again after 15 days and served as its own control. Moose were anesthetized with medetomidine (0.05–0.08 mg kg^−1^), ketamine (1.7–2.8 mg kg^−1^), and butorphanol (0.04–0.07 mg kg^−1^) intramuscularly (IM). Intranasal oxygen (1 L min^−1^ 100 kg^−1^) was administered, and they were allocated to receive either 10 mL saline (control) or aerosolized salbutamol. Salbutamol was administered incrementally at 200, 400 and 800 µg 100 kg^−1^, with 10-minute monitoring intervals following each dose, using one of three methods: a spacer, an equine intranasal (IN) tube, or an equine medical mask. Invasive blood pressure, electrocardiogram, respiratory rate, SpO2, and rectal temperature were recorded every 5 min. Arterial blood gases and electrolytes were measured before oxygen supplementation and both before and 10 min after each salbutamol dose. Anesthesia was reversed with IM atipamezole (0.3–0.4 mg kg^−1^), and all moose were monitored for seven days post-anesthesia for adverse effects. All moose were hypoxemic, hypercapnic, hypertensive, and tachypneic. No hypotension or hypokalemia occurred. PaO₂ increased in all individuals, with no consistent difference between treatment and control. The largest PaO₂ increase occurred in the moose receiving salbutamol via the IN tube. Other physiological variables remained stable. Recoveries were uneventful, with no adverse effects observed.

**Conclusion:**

Aerosolized salbutamol was well tolerated, but did not provide a clear benefit for hypoxemia compared to placebo. Further studies with higher salbutamol doses, alternative delivery routes, or modified anesthetic protocols are recommended.

**Supplementary Information:**

The online version contains supplementary material available at 10.1186/s13028-026-00856-7.

## Background

Due to their reversible and synergistic sedative properties, opioids and α_2_-adrenoceptor (α_2_) agonists are often included in the anesthetic protocol for European moose (*Alces alces alces*), both in captivity and in the wild, where chemical immobilization is required for management, conservation, and research. Their combination improves analgesic efficacy and reduces the required sedative doses for each drug, minimizing the adverse effects and increasing the overall therapeutic index [1]. However, this drug combination can cause severe respiratory compromise and ventilation-perfusion (V/Q) mismatch, resulting in hypoxemia, hypercapnia, and acidosis [2, 3], thereby making medical oxygen supplementation essential.

Salbutamol, a selective β_2_-adrenoreceptor agonist, has been shown to alleviate hypoxemia within 10–15 min in horses anesthetized with drug protocols containing opioids and α_2_-agonists, presumably by reducing V/Q mismatch, when administered at 1–2 µg kg^−1^ via a metered dose inhaler (MDI) connected to the endotracheal tube [4, 5]. Compared with formoterol and salmeterol, which possess adequate lipophilic properties, salbutamol is hydrophilic and therefore has a rapid onset and short duration of action [6], making it suitable for short-term intra-anesthetic interventions. Transient yet significant systemic adverse effects such as hypotension, tachycardia, sweating, and hypokalemia have occasionally been reported [7, 8]. In equine practice, salbutamol is routinely administered to awake horses using spacers or equine fitted masks, specifically designed for delivery of nebulized drugs at recommended doses (2 µg kg^−1^) [9].

The administration of salbutamol could elicit similar beneficial effects in anesthetized moose, thereby potentially alleviating hypoxemia. This could reduce supplemental oxygen requirements and systemic hypertension, which is reported in cervids when using α_2_-agonist-based anesthetic protocols [10]. The aim of this randomized, semi-crossover, controlled case series is to report the effects of three incremental doses of aerosolized salbutamol administered via three different delivery methods feasible for field anesthesia on the physiology of European moose anesthetized with medetomidine-ketamine-butorphanol. This is the first reported case series on the therapeutic use of inhalant salbutamol in anesthetized moose.

## Case presentation

Two juvenile (female and male, both 10 months old, case study 1 and 2, respectively) and one adult (male, 6 years old, case study 3) moose were included in the clinical trial during pre-scheduled anesthesia for routine health assessments at Bern Animal Park, Switzerland. The juvenile moose from case 1 was anesthetized again 15 days later for transport to a different zoological institution and was included in the trial as a control (case study 4). All moose were deemed healthy prior to anesthesia and were separated into individual enclosures one hour before the procedure. They were darted via a CO_2_-powered dart-gun (DanInject, Børkop, Denmark) with a combination of approximately 0.06 mg/kg of medetomidine (Medetomidine, Christoffel Apotheke, Bern, Switzerland; 20 mg mL^−1^), 2 mg kg^−1^ of ketamine (Ketamine, Christoffel Apotheke, Bern, Switzerland; 166 mg mL^−1^), and 0.05 mg kg^−1^ of butorphanol (Morphasol, Dr. E Graeub AG, Bern, Switzerland; 10 mg mL^−1^) based on estimated bodyweight.

Once the moose were sufficiently anesthetized and blindfolded, arterial blood was aseptically collected from the caudal auricular artery for immediate baseline blood-gas and electrolyte analysis using a portable analyzer (CG8+, i-STAT, Abbott, Abbott Park, Illinois, USA). Blood gas and pH values were corrected for rectal temperature at the time of sampling. Alveolar oxygen tension (P_A_O_2_) and alveolar-arterial oxygen tension gradient (P_(A−a)_O_2_) were calculated as previously described in moose [2]. Immediately after the initial arterial sample, medical oxygen was supplemented at 1 L minute^−1^ 100 kg^−1^ using an oxygen cylinder (Carbagas AG, Gümligen, Switzerland) via a cannula inserted 10 cm into the left nasal cavity. The animals were then weighed and, if initial body weight had been underestimated, the remaining calculated drug dose was administered intramuscularly (IM). All moose were positioned in sternal recumbency and instrumented with a multiparameter monitor (Datalys V5, Lutech Medical, Ronkonkoma, New York, USA) for continuous monitoring of physiological parameters (blood pressure, electrocardiogram, rectal temperature, peripheral oxygen saturation, respiratory rate). A 20G catheter was aseptically placed in the caudal auricular artery and connected to a heparinized (Heparin sodium, Wockhardt, Wrexham, UK, 1000 IU mL^−1^; diluted to 5 IU mL^−1^) arterial line for invasive blood pressure (IBP) monitoring and arterial blood sampling. The pressure transducer was positioned at the level of the right atrium, zeroed to atmospheric pressure, and a dynamic response test was performed to confirm absence of damping artifact.

**Fig. 1:**
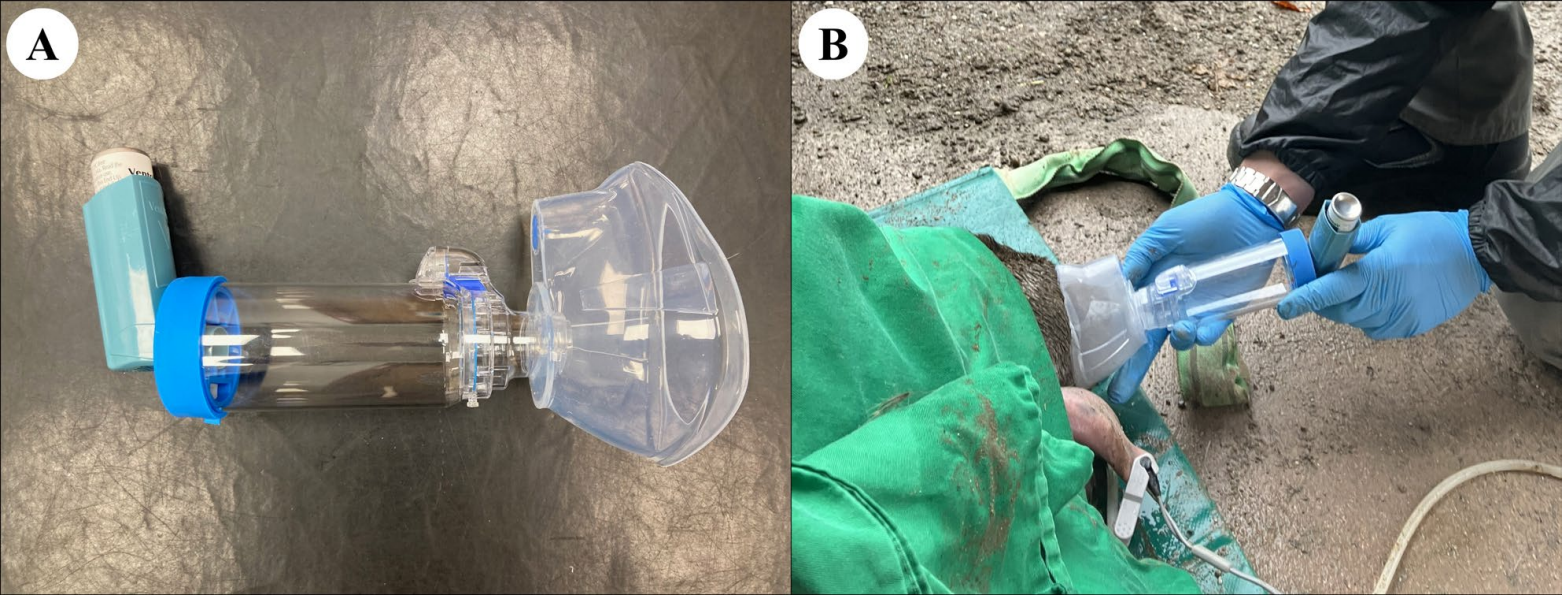
**A**, **B** Spacer method. Photographic representations of the metered dose inhaler (MDI) connected to the spacer used in Case Study 1. Oxygen is supplemented via a cannula inserted in the left nostril

**Fig. 2:**
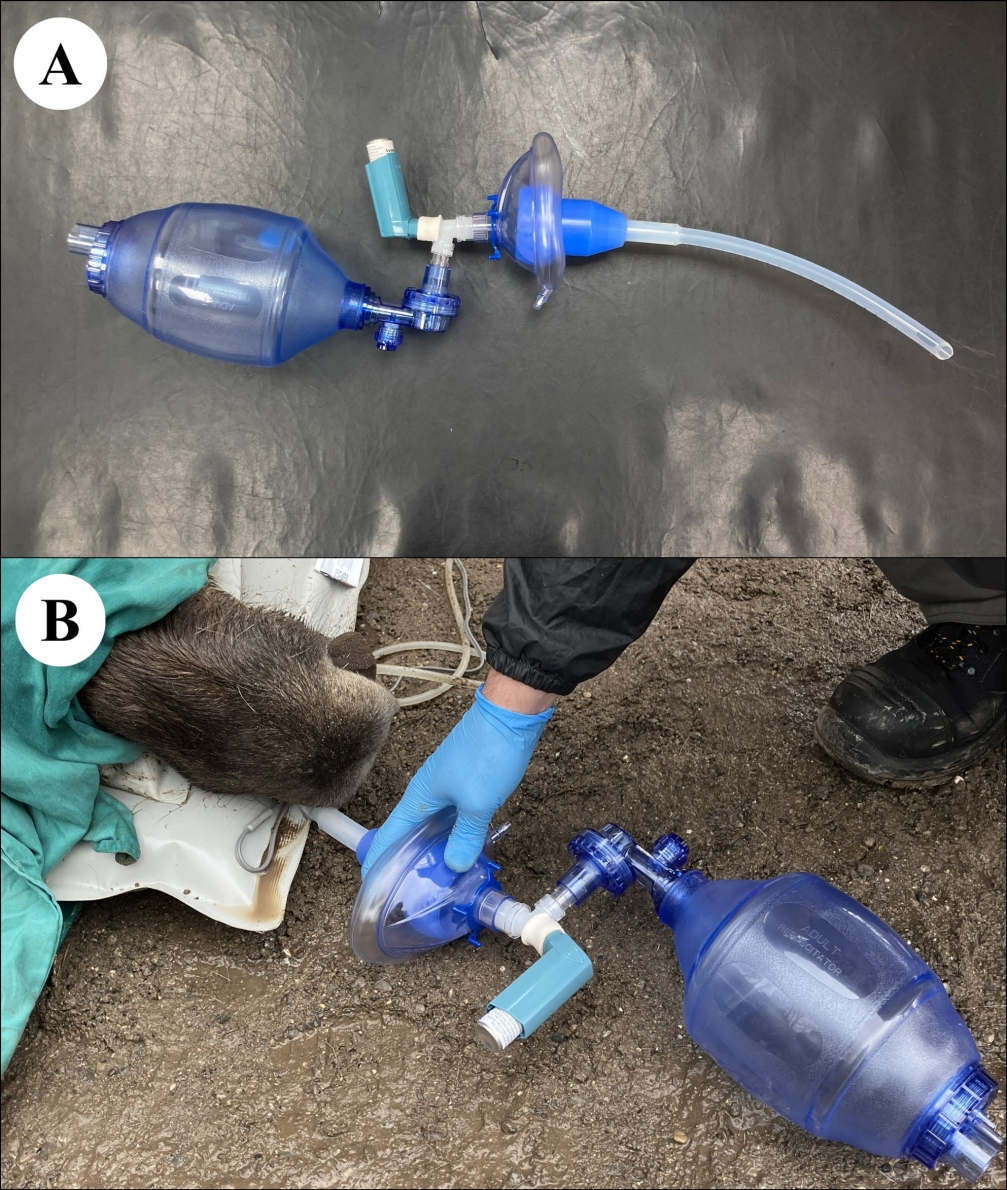
**A**, **B** Intranasal method. Photographic representations of the MDI connected to the equine intranasal (IN) tube used in Case Study 2. Oxygen is supplemented via a cannula inserted in the left nostril

**Fig. 3:**
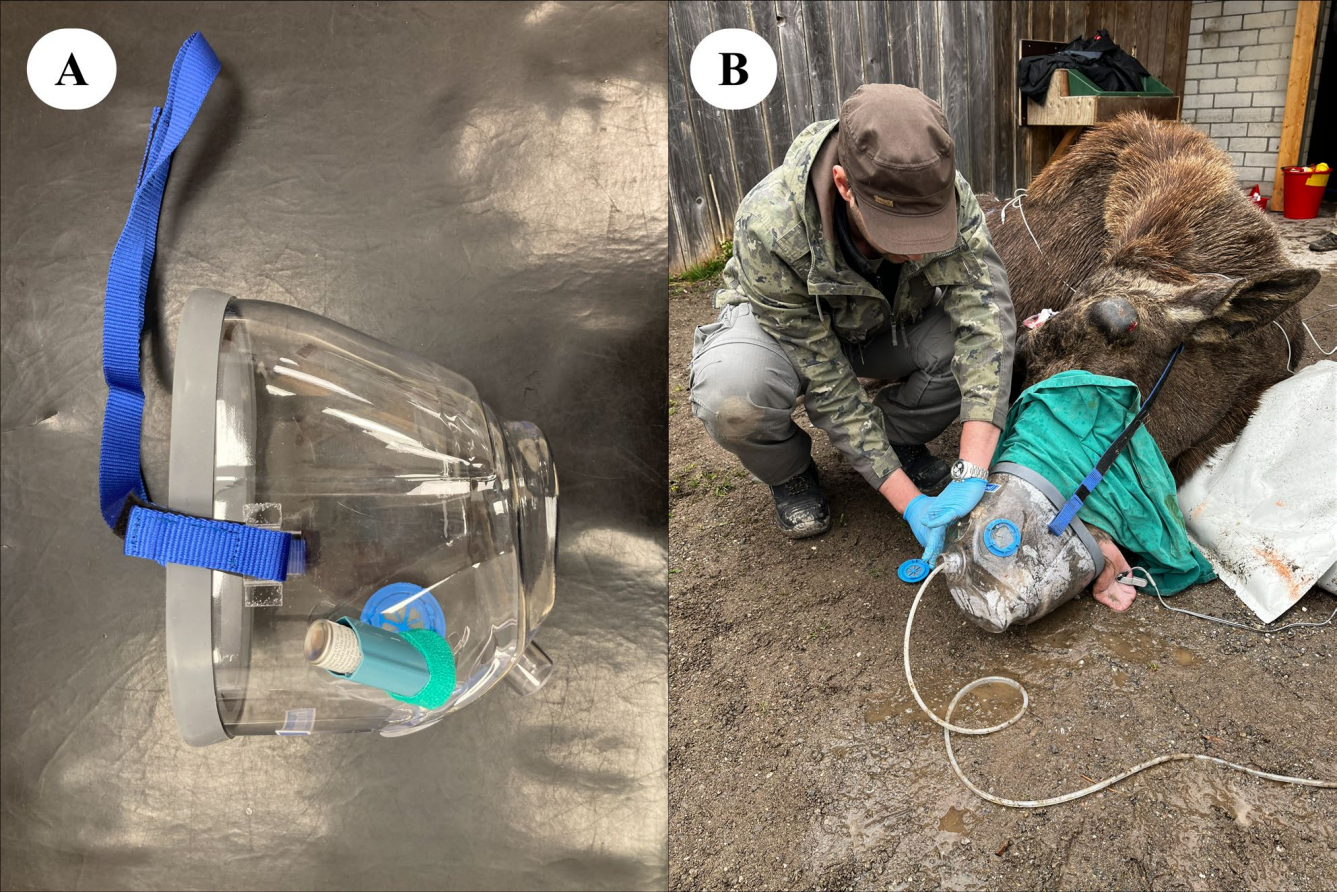
**A**, **B** Mask method. Photographic representations of the MDI connected to the equine inhaler mask used in Case Study 3. Oxygen is supplemented via a cannula inserted in the left nostril

Each moose was randomly allocated to either placebo (case 4; 10 mL of sterile 0.9% NaCl injected IM), or one of three salbutamol (Ventolin Evohaler inhaler, GlaxoSmithKline, London, UK, 100 ug per metered actuation) delivery methods: either connection of the MDI with a spacer (case 1; Comfort Chamber, Ved Healthcare, Watford, UK), an equine silicone intranasal (IN) tube (case 2; Equine recovery tube, Surgite Limited, Newport Pagnell, UK), or an equine inhaler mask (case 3; Coldblood Inhaler mask, Hippomed, Cityville USA). Approximately 20 min after initiating oxygen supplementation, a second arterial blood sample was collected and analyzed and then salbutamol was administered at 200 µg 100 kg^−1^ via the allocated delivery method. In case 1, the silicon mask at one extremity of the spacer was tightly placed over the right nostril and was left in place during the metered actuations and for the following 2 min to maximize salbutamol inhalation. In case 2, the IN tube was lubricated, inserted 25 cm into the right ventral meatus, and remained in place for the duration of anesthesia. The MDI was connected to the IN tube and a 1.5-L bag valve mask (Manual Resuscitator, Reflex Medical, Shepton Mallet, UK) via an adapted Y-connector, so that each metered actuation was flushed down the IN tube by simultaneously compressing the bag during each inspiratory phase. In case 3, the equine mask was tightly secured around the muzzle and remained in place for the entire procedure. In the latter case, the MDI was connected to the inspiratory valve in front of the right nostril as previously described [11] only at the time of salbutamol administration. The oxygen cannula was maintained in the left nostril to standardize supplementation across the anesthetic events. Case 4 (control moose) was also equipped with the IN tube in the right nostril. The three methods of administration are presented in Figs. 1, 2 and 3 and in the Additional Files 1–3.

Ten minutes after the first salbutamol dose, a third arterial blood sample was analyzed, and therapeutic and adverse effects were assessed. If serum potassium and mean arterial pressure (MAP) were above the safety thresholds of 3.0 mmol L^−1^ for potassium and 70 mmHg for MAP, a doubled salbutamol dose (400 µg 100 kg^−1^) was administered. Subsequently, if no hypokalemia or hypotension occurred after a further 10 min, a final dose of 800 µg 100 kg^−1^ was administered, followed by a final arterial blood sample after the same time interval. In cases of hypokalemia or hypotension, an infusion of potassium chloride (Kaliumchlorid, B. Braun, Medical, Switzerland; 14.9%) at 0.25 mmol kg^−1^ h^−1^ or dobutamine (DOBUTamine, Hameln Pharma, Gloucester, UK; 12.5 mg mL^−1^) at 1 µg kg^−1^ minute^−1^ was initiated, respectively.

At the end of each procedure, medetomidine was reversed with atipamezole (Alzane ad us. vet., Dr. E. Graeub AG, Bern, Switzerland; 5.0 mg mL^−1^), an α_2_-antagonist, at 5 mg per mg of medetomidine to reverse immobilization. Quality and time of recovery were recorded. All moose were monitored for 7 days, and any post-anesthetic adverse effects were documented.

Data analysis, descriptive statistics, graphs and visualization of the data were performed in Microsoft Excel (version 2311). No advanced statistical analyses could be performed as a result of the heterogeneity and limited sample size of the presented case series. All moose achieved sternal recumbency rapidly following darting (median induction time 5 (range 5–10) minutes), with induction quality was considered good. Actual median doses of medetomidine, ketamine and butorphanol were 0.06 (range 0.05–0.08) mg kg^−1^, 1.9 (range 1.7–2.8) mg kg^−1^, and 0.05 (range 0.04–0.07) mg kg^−1^, respectively. At the time of the first arterial blood sample, prior to oxygen supplementation (median time from darting 15 min; range 12–27 min), all moose were hypercapnic (median P_a_CO_2_ 52 mmHg; range 54–66 mmHg), and hypoxemic (median P_a_O_2_ 47 mmHg; range 41–70 mmHg), with impaired oxygen exchange, indicated by elevated P_(A−a)_O_2_ (median 38 mmHg; range 29–47 mmHg). Potassium concentrations were within the normal range (median potassium 4.0 mmol L^−1^; range 3.7–4.5 mmol L^−1^). Three out of four moose (cases 1, 2 and 3) developed mild to moderate respiratory acidosis, persisting in two individuals (cases 2 and 3) until the end of the monitoring period.

The median cumulative salbutamol dose administered was 14.5 (range 12.2–15.0) µg kg^−1^. No adverse effects, such as hypotension, hypokalemia, tachycardia or arrhythmias, were observed following the three salbutamol administrations.

P_a_O_2_, S_p_O_2_, and S_a_O_2_ steadily increased over time in all moose regardless of the method of salbutamol administration or placebo. The two juveniles fitted with an IN tube, one receiving salbutamol and one placebo (cases 2 and 4), had slightly higher P_a_O_2_ by the end of the monitoring period (123–124 mmHg vs. 100–108 mmHg). Moreover, the highest P_a_O_2_ increase from baseline to the final arterial sample occurred in the moose receiving salbutamol via the IN tube (case 2), rising by 80 mmHg compared to 54–59 mmHg in the others.

**Fig. 4:**
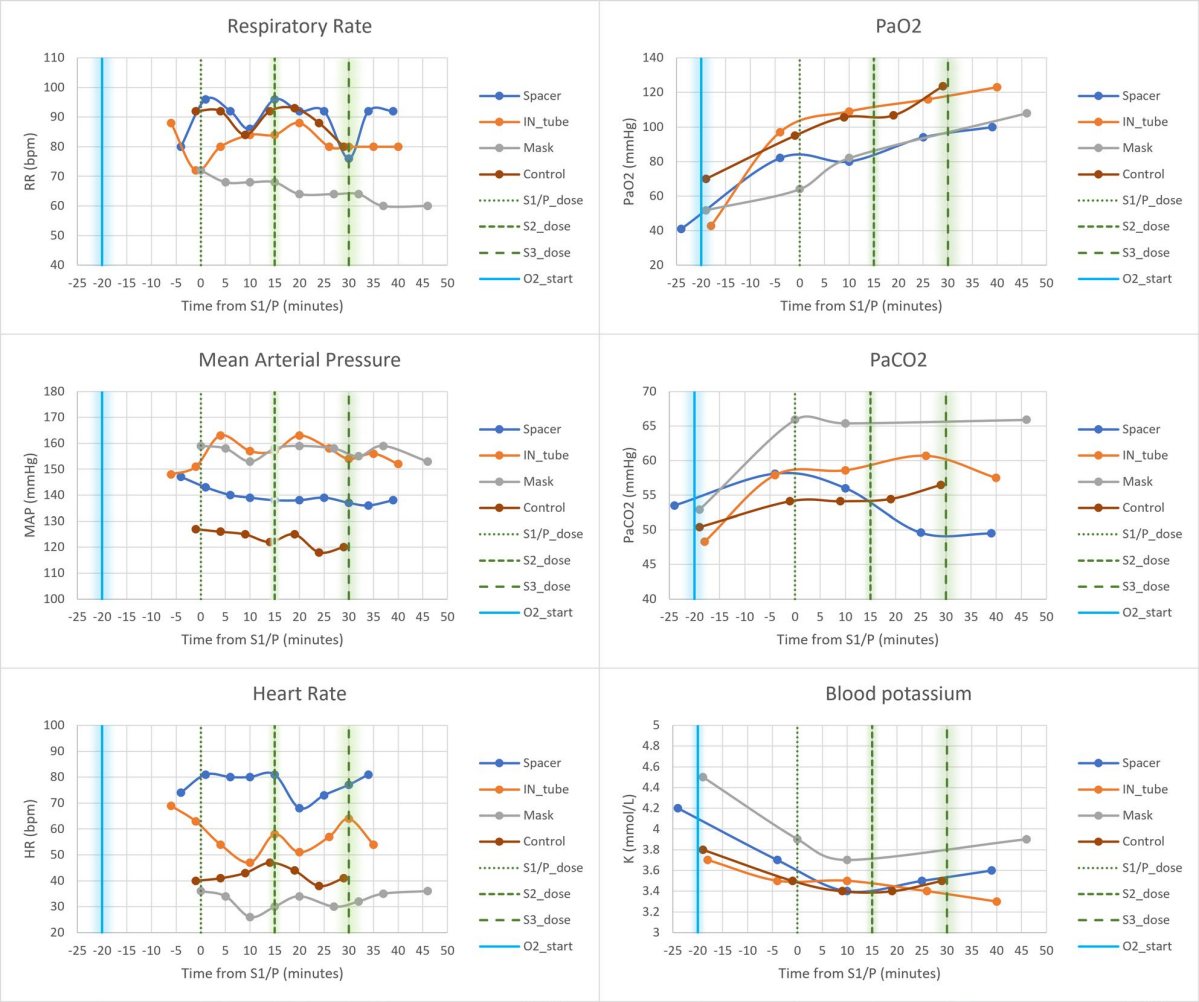
Physiological effects of incremental salbutamol doses across the case series. Main physiological variables measured at regular intervals during the anesthesia of three captive European moose. The moose allocated to the spacer (Case 1) was anesthetized again and used as a control (Case 4). The median (with shadowed range) start time of oxygen supplementation is represented as a vertical blue straight line. The median (with shadowed range) times of administration of salbutamol are represented as vertical green dashed lines. Placebo was administered at the same time as S1 in the control moose. Abbreviations: S1,2,3 = 1st, 2nd, 3rd salbutamol administration; *P *placebo administration, *IN*  intranasal, *Ma *mask, *C *control, HR heart rate, *RR* respiratory rate, *MAP* mean arterial pressure, *PaCO*_*2*_  arterial partial pressure of carbon dioxide, *PaO*_*2*_  arterial partial pressure of oxygen, *K* potassium

Although tachypneic, all animals remained hypercapnic, with the adult moose (case 3) exhibiting the highest P_a_CO_2_ (66 mmHg). All moose were hypertensive, maintained a regular sinus rhythm, and had a stable heart rate throughout anesthesia, with higher heart rates in juveniles than the adult. Case 2 developed mild hyperthermia (40.2 °C). Other physiological variables were unremarkable. More details of the main physiological variables are presented in Table 1; Fig. 4, with more extensive data available in the Additional File 4.

**Table 1:** Main physiological variables measured at regular intervals during the anesthesia of three captive European moose

Median time from S1/*P* (min) ►		-35	-20	0	5	10	15	20	25	30	35	40
Events ►		Darting	pre-O_2_ ABG	S1/*P* + ABG		ABG	S2		ABG	S3		ABG
HR (bpm)	Case 1 (Sp)	-	-	74	81	80	80	81	68	73	77	81
	Case 2 (INt)	-	-	69	63	54	47	58	51	57	64	54
	Case 3 (Ma)	-	-	36	34	26	30	34	30	32	35	36
	Case 4 (C)	-	-	40	41	43	47	44	38	41	40	-
RR (bpm)	Case 1 (Sp)	-	-	80	92	86	96	92	92	76	92	92
	Case 2 (INt)	-	-	72	80	84	84	88	80	80	80	80
	Case 3 (Ma)	-	-	72	68	68	68	64	64	64	60	60
	Case 4 (C)	-	-	92	92	84	92	93	88	80	-	-
MAP (mmHg)	Case 1 (Sp)	-	-	147	140	139	138	138	139	137	136	138
	Case 2 (INt)	-	-	151	163	157	157	163	158	154	156	152
	Case 3 (Ma)	-	-	159	158	153	158	159	158	155	159	153
	Case 4 (C)	-	-	127	126	125	122	125	118	120	-	-
RT (° C)	Case 1 (Sp)	-	-	39.1	39	39.1	39.1	39	38.9	38.9	38.9	38.9
	Case 2 (INt)	-	-	40.2	40.1	40.2	40.2	40.1	40	39.9	39.8	39.7
	Case 3 (Ma)	-	-	38.6	38.7	38.7	38.6	38.7	38.7	38.6	38.6	38.6
	Case 4 (C)	-	-	39	39	38.9	38.9	38.9	38.9	38.8	38.8	-
pH (U)	Case 1 (Sp)	-	7.353	7.322	-	7.329	-	-	7.410	-	-	7.402
	Case 2 (INt)	-	7.348	7.288	-	7.306	-	-	7.317	-	-	7.341
	Case 3 (Ma)	-	7.335	7.236	-	7.246	-	-	-	-	-	7.288
	Case 4 (C)	-	7.372	7.359	-	7.377	-	-	7.389	-	-	7.380
P_a_CO_2_ (mmHg)	Case 1 (Sp)	-	54	58	-	56	-	-	50	-	-	50
	Case 2 (INt)	-	48	58	-	59	-	-	61	-	-	58
	Case 3 (Ma)	-	53	66	-	65	-	-	-	-	-	66
	Case 4 (C)	-	50	54	-	54	-	-	54	-	-	56
P_a_O_2_ (mmHg)	Case 1 (Sp)	-	41	82	-	80	-	-	94	-	-	100
	Case 2 (INt)	-	43	97	-	109	-	-	116	-	-	123
	Case 3 (Ma)	-	52	64	-	82	-	-	-	-	-	108
	Case 4 (C)	-	70	95	-	106	-	-	107	-	-	124
S_a_O_2_ (%)	Case 1 (Sp)	-	65	93	-	92	-	-	96	-	-	97
	Case 2 (INt)	-	64	94	-	96	-	-	97	-	-	98
	Case 3 (Ma)	-	79	84	-	92	-	-	-	-	-	97
	Case 4 (C)	-	91	96	-	97	-	-	97	-	-	98
K (mmol/L)	Case 1 (Sp)	-	4.2	3.7	-	3.4	-	-	3.5	-	-	3.6
	Case 2 (INt)	-	3.7	3.5	-	3.5	-	-	3.4	-	-	3.3
	Case 3 (Ma)	-	4.5	3.9	-	3.7	-	-	-	-	-	3.9
	Case 4 (C)	-	3.8	3.5	-	3.4	-	-	3.4	-	-	3.5

The moose allocated to the spacer (Case 1) was anesthetized again and used as a control (Case 4). *S1*,*2*,*3* = 1st, 2nd, 3rd salbutamol administration, *P* placebo administration, *min* minutes, *ABG* arterial blood-gas analysis, *Sp* spacer, *INt* intranasal tube, *Ma* mask, *C* control, *HR* heart rate, *RR* respiratory rate, *MAP* mean arterial pressure, *RT* rectal temperature, *P*_*a*_*CO*_*2*_ arterial partial pressure of carbon dioxide, *P*_*a*_*O*_*2*_ arterial partial pressure of oxygen, *S*_*a*_*O*_*2*_ arterial oxygen saturation, *K* potassium

The median duration of anesthesia was 76 (range 68–78) minutes. Recovery was steady (median time from reversal to standing 16 min; range 12–20 min) and smooth following atipamezole administration (median 0.39 mg kg^−1^; range 0.31–0.42 mg kg^−1^). No adverse effects were observed during the 7-day post-anesthetic monitoring period.

## Discussion and conclusions

The results of the pre-oxygenation blood samples reflect the well-recognized respiratory effects of opioid and α_2_-agonists in moose, including dead-space ventilation, alveolar hypoventilation, increased diffusion barrier, V/Q mismatch, and increased physiological shunt [2]. These mechanisms impair pulmonary gas exchange and contribute to hypoxemia, for which oxygen supplementation is beneficial.

The lack of a clinically significant difference in P_a_O_2_ between treatment and control groups is likely multifactorial. Potential contributors include suboptimal delivery methods for this species or respiratory pattern (i.e., tachypnoea and shallow breathing), inefficacy at the reported MKB doses, milder hypoxemia in the control moose compared to treated individuals, the confounding effects of concurrent oxygen supplementation, and possible absorption of the drug via the naso-oro-gastric mucosa. Although the cumulative salbutamol dose in the current trial was seven-fold the recommended dose for spacers and masks in unanesthetized horses [9], only 53% of the aerosolized salbutamol is reported to reach the equine airways when using a spacer [11]. Additionally, the proportion reaching the alveoli is likely even lower in anesthetized moose, due to the abundant soft tissue present in the nasal cavities [12] and the clinical status specific to MKB anesthesia (e.g., tachypnoea, alveolar hypoventilation, interstitial pulmonary oedema, etc.).

As no hypokalemia, tachyarrhythmias, hypotension, or other adverse effects attributable to salbutamol were observed, a higher dose could potentially be administered safely to achieve clinically effective pulmonary concentrations. However, in large animals, such as moose, delivering multiple 100-µg-actuations synchronized with consecutive inspiratory cycles is logistically challenging, and increasing the number of actuations does not proportionally increase the amount of drug available for inhalation [13]. While endotracheal intubation may enhance drug delivery efficiency [4], it was not considered in this study, as the goal was to assess a field-applicable protocol in which moose are not routinely intubated. Although intravenous salbutamol administration is more practical, this route has been associated with limited efficacy or even worsening of V/Q mismatch, due to vasodilation of poorly ventilated lung regions [14].

The lower respiratory rate and the larger body size of the bull compared to the juveniles could have contributed to a higher risk of hypoventilation and pulmonary atelectasis [15, 16], potentially contributing to the more pronounced respiratory acidosis [17]. An influence of the mask, however, cannot be excluded. In exercising horses, masks equipped with outflow valves are reported to alter blood gas parameters compared with open-flow masks, due to increased resistance and rebreathing of expired air [18]. However, such changes were not significant in resting animals, and similar effects have not been demonstrated in anesthetized horses. Interestingly, a recent study demonstrated that anesthetized horses equipped with a facemask recruited more alveoli and increased tidal impedance variation compared to endotracheally intubated horses in spontaneous ventilation [19].

This case series had several limitations that should be considered when interpreting the findings, including a small sample size and variability in age and body size, which prevented a comprehensive data analysis and could have affected the response to both MKB and salbutamol. Supplemental oxygen played a confounding role in the assessment of the effect of salbutamol on the V/Q mismatch; however, its use was warranted from an ethical and professional standpoint. In addition, the tachypnoea and dead-space ventilation seen in all moose of this case series could have decreased the MDI delivery success either by not delivering a clinically effective dose of salbutamol in the lower airways or by forcing it out of the delivery systems through the safety one-way valves during frequent exhalations, or both. Finally, the blood salbutamol concentration could not be measured at the time of the anesthetic events, preventing a complete assessment of the absorbed fraction of salbutamol and the pharmacokinetic profile of each delivery method.

In conclusion, administration of salbutamol via the routes and dosages recommended in equine medicine did not result in clinically significant benefits or adverse effects in anesthetized captive moose compared to placebo, regardless of the delivery method. All moose exhibited hypoxemia and hypercapnia, with two remaining acidotic throughout the procedure. Oxygen supplementation effectively improved oxygenation, with the highest P_a_O_2_ increase observed in the individual equipped with the IN tube. Further studies involving a larger sample size, higher salbutamol doses, alternative delivery methods or bronchodilators, or modified anesthetic protocols are recommended to assess the feasibility and efficacy of salbutamol administration during field anesthesia in moose.

## Supplementary Information

Additional file 1. Short demonstrative video of the administration of salbutamol via the spacer. Oxygen is supplemented via a cannula inserted in the left nostril.

Additional file 2. Short demonstrative video of the administration of salbutamol via the IN tube. Oxygen is supplemented via a cannula inserted in the left nostril.

Additional file 3. Short demonstrative video of the administration of salbutamol via the mask. Oxygen is supplemented via a cannula inserted in the left nostril.

Additional file 4. Detailed spreadsheets with times, drug doses, physiological variables, and arterial blood results of the four moose anesthetized during the clinical trial.

## Data Availability

The original datasets used, analyzed and presented in the current case series and additional files are available from the corresponding author on reasonable request.

## Abbreviations

IBP: Invasive blood pressure
IN: Intranasal
MAP: Mean arterial pressure
MDI: Metered dose inhaler
MKB: Medetomidine-ketamine-butorphanol
PaCO_2_: Partial pressure of arterial carbon dioxide
PaO_2_: Partial pressure of arterial oxygen
P_A_O_2_: Alveolar oxygen tension
P_(A−a)_O_2_: Alveolar-arterial oxygen tension gradient
SaO_2_: Arterial hemoglobin-oxygen saturation
SpO_2_: Peripheral hemoglobin-oxygen saturation
V/Q: Ventilation/perfusion

## Declarations

### Prior publication

Data have not been published previously.
